# Supplementary material for: Identification and characterisation of pathogenic and non-pathogenic FGF14 repeat expansions
Source: Nat Commun. 2024 Sep 3;15:7665. doi: 10.1038/s41467-024-52148-1 (PMC11372089; doi:10.1038/s41467-024-52148-1)
Supplement: Supplementary file 1 — Supplementary Information [file 41467_2024_52148_MOESM1_ESM.pdf]

Identification and characterisation of pathogenic and non-pathogenic *FGF14* repeat expansions

Lars Mohren[1,20], Friedrich Erdlenbruch[2,20], Elsa Leitão[1,20], Fabian Kilpert[1,20], G. Sebastian Hönes[3], Sabine Kaya[1], Christopher Schröder[1], Andreas Thieme[2], Marc Sturm[4], Joohyun Park[4], Agatha Schlüter[5,6], Montserrat Ruiz[5,6], Moisés Morales de la Prida[5,7], Carlos Casasnovas[5,6,7], Kerstin Becker[8], Ulla Roggenbuck[9], Sonali Pechlivanis[9,10], Frank J. Kaiser[1,11], Matthis Synofzik[12,13], Thomas Wirth[14,15,16], Mathieu Anheim[14,15,16], Tobias B. Haack[4,17], Paul J. Lockhart[18], Karl-Heinz Jöckel[9], Aurora Pujol[5,6,19], Stephan Klebe[2], Dagmar Timmann[2], Christel Depienne[1*]

Author affiliations:

[1] Institute of Human Genetics, University Hospital Essen, University Duisburg-Essen, Essen, Germany. [2] Department of Neurology and Center for Translational Neuro- and Behavioral Sciences (C-TNBS), University Hospital Essen, University Duisburg-Essen, Essen, Germany. [3] Department of Endocrinology, Diabetes and Metabolism, University Hospital Essen, University of Duisburg-Essen, Essen, Germany. [4] Institute of Medical Genetics and Applied Genomics, University of Tübingen, Tübingen, Germany. [5] Neurometabolic Diseases Laboratory, Bellvitge Biomedical Research Institute (IDIBELL), Barcelona, Spain. [6] CIBERER, Centro de Investigación Biomédica en Red de Enfermedades Raras, ISCIII, Madrid, Spain. [7] Neuromuscular Unit, Neurology Department, Bellvitge University Hospital, Barcelona, Spain. [8] Cologne Center for Genomics (CCG), University of Cologne, Faculty of Medicine and University Hospital Cologne, 50931 Cologne, Germany. [9] Institute for Medical Informatics, Biometry and Epidemiology, University Hospital Essen, University of Duisburg-Essen, Essen, Germany. [10] Institute of Asthma and Allergy Prevention, Helmholtz Zentrum München, German Research Center for Environmental Health, Neuherberg, Germany . [11] Essener Zentrum für Seltene Erkrankungen (EZSE), Universitätsklinikum Essen, Essen, Germany. [12] Division Translational Genomics of Neurodegenerative Diseases, Center for Neurology & Hertie Institute for Clinical Brain Research Tübingen, Germany. [13] German Center for Neurodegenerative Diseases (DZNE), Tübingen, Germany. [14] Service de Neurologie, Département de Neurologie, Hôpitaux Universitaires de Strasbourg, Hôpital de Hautepierre, 1, Avenue Molière, 67098, Strasbourg Cedex, France. [15] Institut de Génétique et de Biologie Moléculaire et Cellulaire (IGBMC), INSERM-U964/CNRS-UMR7104/Université de Strasbourg, Illkirch, France. [16] Fédération de Médecine Translationnelle de Strasbourg (FMTS), Université de Strasbourg, Strasbourg, France. [17] Centre for Rare Diseases, University of Tübingen, Tübingen, Germany. [18] Bruce Lefroy Centre, Murdoch Children's Research Institute; Department of Paediatrics, The University of Melbourne, Parkville, VIC, Australia. [19] Catalan Institution of Research and Advanced Studies (ICREA), Barcelona, Spain. [20] These authors contributed equally: Lars Mohren, Friedrich Erdlenbruch, Elsa Leitão, Fabian Kilpert

Correspondence to: Christel Depienne, Institut für Humangenetik, Universitätsklinikum Essen, Virchowstraße 171, 45147 Essen, Germany. E-mail: christel.depienne@uk-essen.de

Supplementary Figures

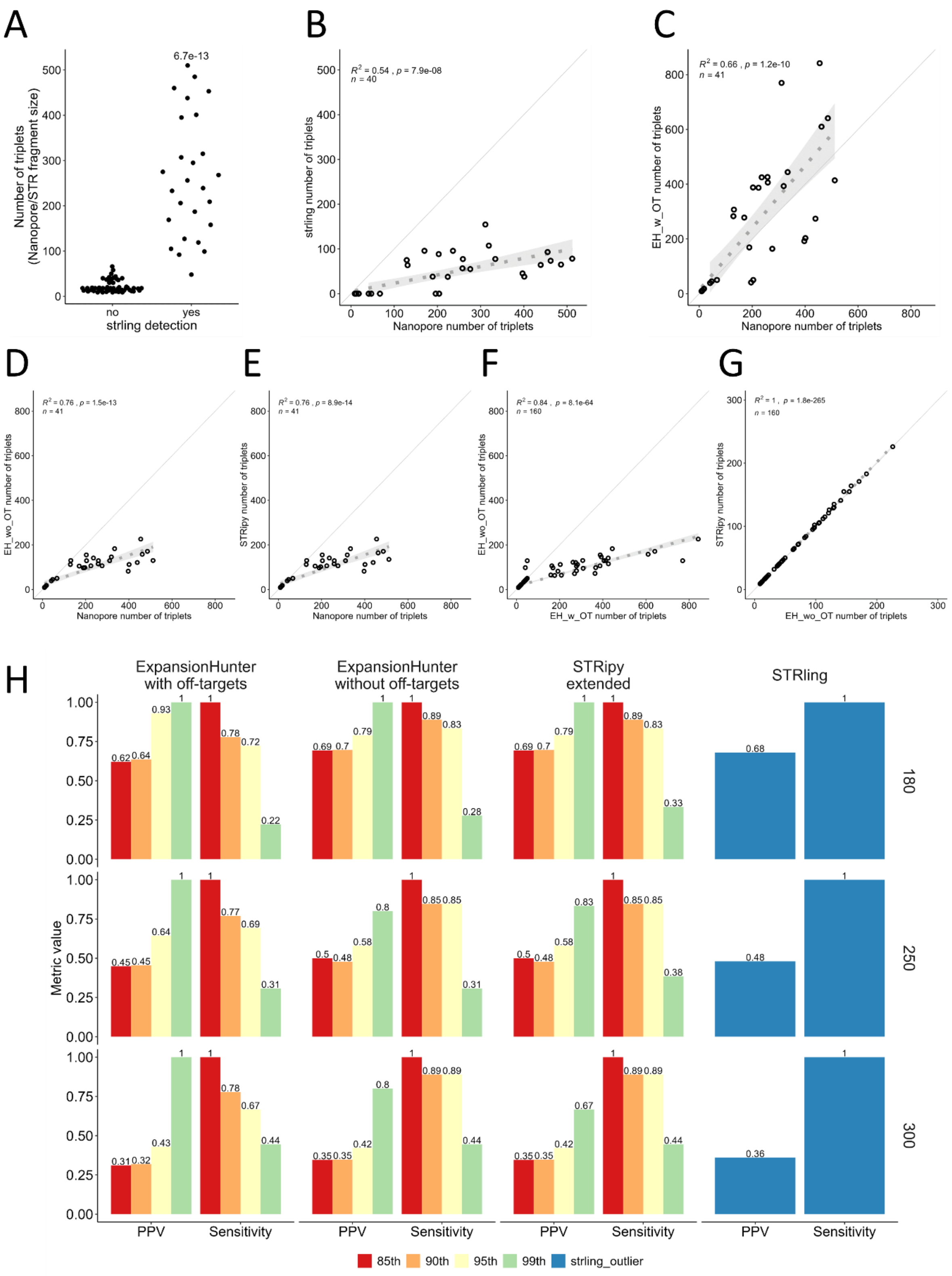

Supplementary Figure 1. **Comparison of the ability of bioinformatics tools to detect *FGF14* repeat expansions and repeat numbers**. **A)** Median number of repeats detected by

nanopore sequencing in samples for which a significant outlier value was (*n*= 26) or not (*n*=54) detected by STRling. **B)** Correlation between the median number of repeats detected by nanopore sequencing and repeat number estimated by STRling. **C)** Correlation between the median number of repeats detected by nanopore sequencing and repeat number estimated by ExpansionHunter (with off targets mode). **D)** Correlation between the median number of repeats detected by nanopore sequencing and repeat number estimated by ExpansionHunter (without off targets mode). **E)** Correlation between the median number of repeats detected by nanopore sequencing and repeat number estimated by STRipy (extended mode). **F)** Correlation between the number of repeats detected by ExpansionHunter with (x axis) or without (y axis) off targets. **G)** Correlation between the number of repeats detected by ExpansionHunter without off target and the repeat number estimated by STRipy (extended mode). For graphs B) to G), R^2 is the square value of the Pearson correlation coefficient (two-sided) and 95% confidence intervals appear in light gray. **H)** Predictive values (PPV) and sensitivity assessed for approaches based on outlier detection using ExpansionHunter with off-targets, ExpansionHunter without off-targets, STRipy (extended mode) and STRling. Quantiles of value distribution were calculated for ExpansionHunter with off-targets, ExpansionHunter without off-targets, and STRipy (extended mode) and different quantiles (85th, 90th, 95th, 99th) were considered for defining outliers whereas outlier detection was based on significant qvalues when using STRling. The results indicate that all tools have a similar sensitivity of 100% when using the 85th percentile (or qvalue for STRling), with predictive values for detecting more than 180 repeats ranging from 62% to 69%. Using upper quantiles increases the predictive values but decreases sensitivity. Number of repeats corresponding to the specific quantiles are in Supplementary Data 8. Each bar represents a single data point. Red: 85th percentile; orange: 90th; yellow: 95th; green: 99th; blue: STRling outlier.

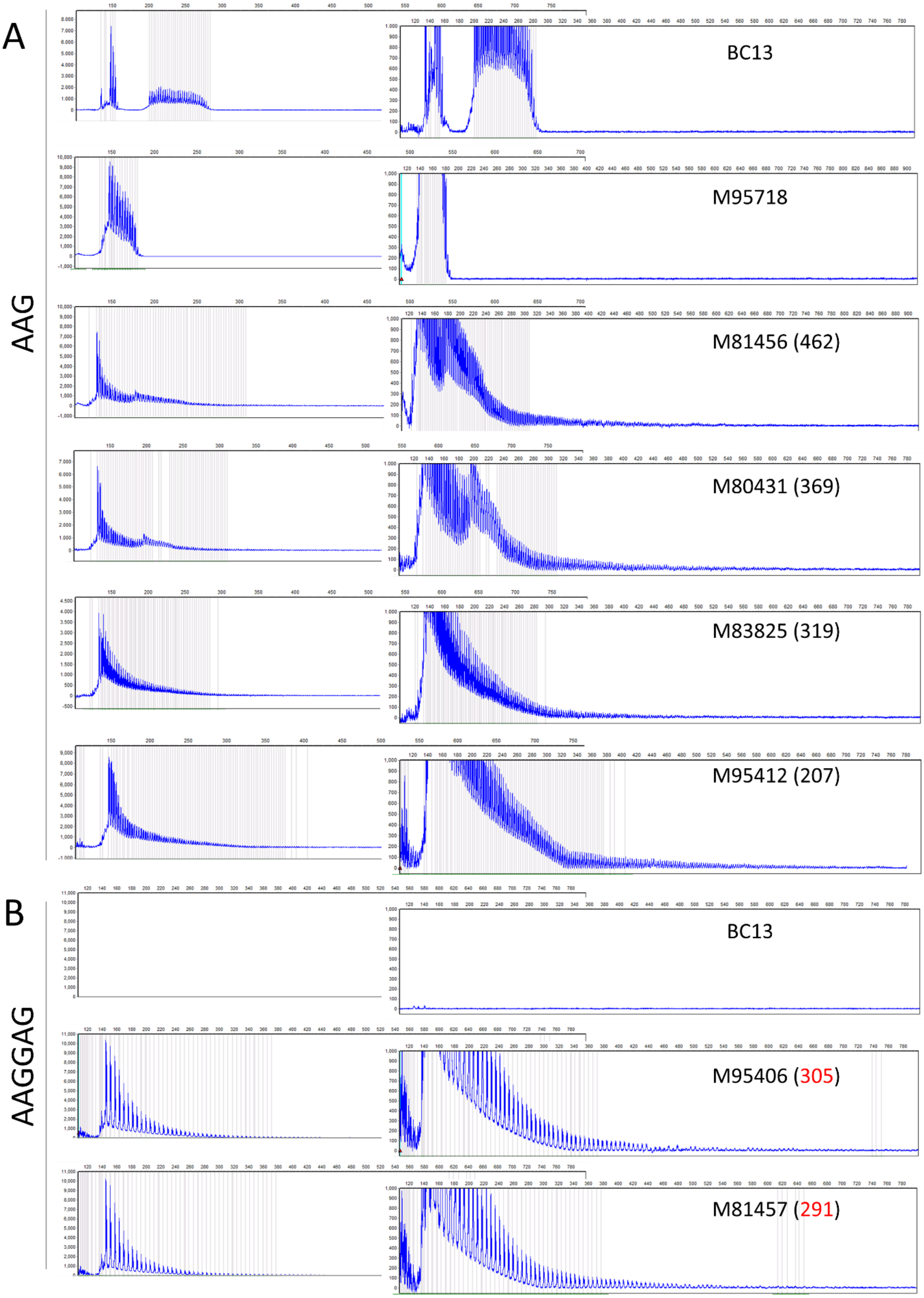

Supplementary Figure 2. Limited ability of repeat-primed PCR (RP-PCR) to distinguish intermediate from pathogenic alleles. A) RP-PCR profiles using AAG primer for a control individual (BC13) and five patients with cerebellar ataxia. M95718 is negative. The other four

patients show a positive profile that cannot be used to distinguish with precision patients with alleles below the pathogenic threshold, intermediate alleles, and pathogenic repeat expansions. The number of expanded AAG repeats appears in brackets. **B)** RP-PCR profiles using AAGGAG primer for a control individual (BC13) and two patients with cerebellar ataxia with an AAGGAG expansion. The red numbers in brackets correspond to the median number of triplets (i.e., AAGGAG would be counted as two triplets) to be directly comparable with AAG repeats. RR-PCR assays were repeated at least twice with the same results.

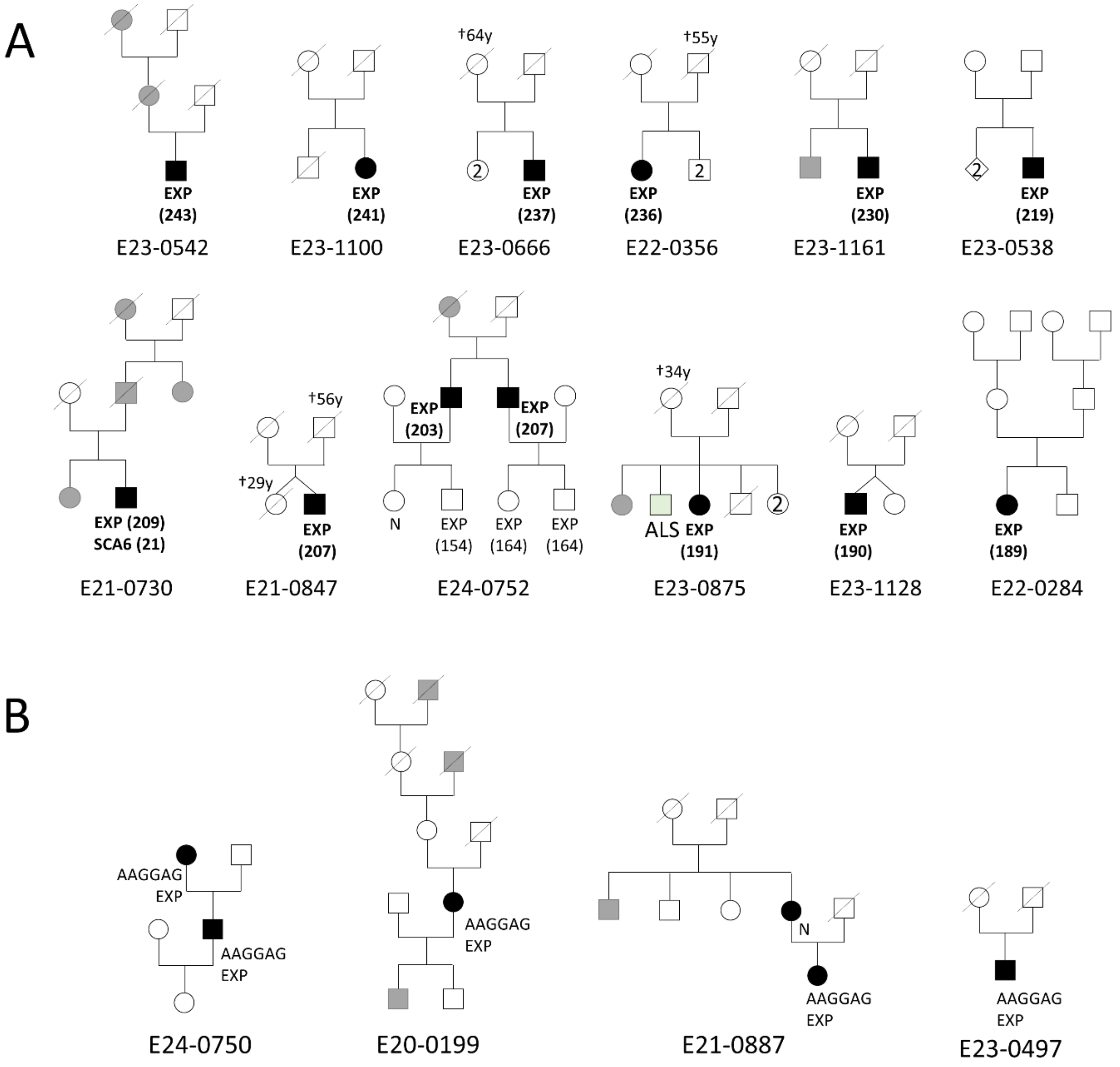

Supplementary Figure 3. **Pedigrees of families with intermediate alleles and AAGGAG repeat expansions. A)** Pedigrees of families with *FGF14* intermediate alleles (number of repeats between 180-249 repeats). **B)** Pedigrees of families with cerebellar ataxia and AAGGAG repeat expansion. Black symbols indicate affected subjects examined and sampled in the study. Gray symbols indicate subjects reported to be affected on history but that could not be examined. The number in brackets indicates the median number of repeats for the affected individuals of this family. The numbers in symbols indicate the number of siblings with the same sex. N: normal (no expansion); AAGGAG EXP: AAGGAG hexameric expansion; EXP: AAG expansions.

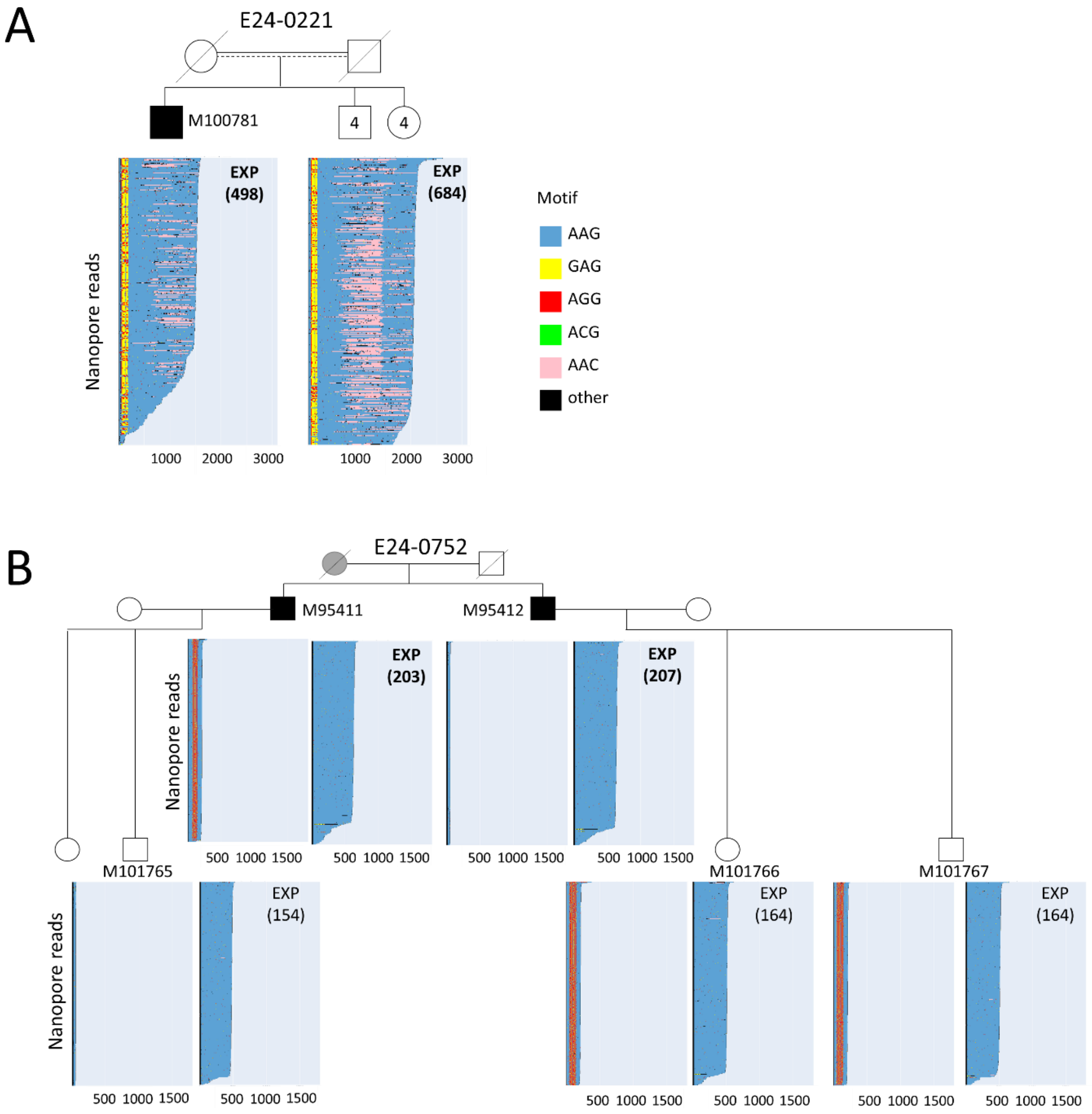

Supplementary Figure 4. **Pedigrees of families with intermediate alleles and AAGGAG repeat expansions. A)** Schematic representation of nanopore reads sequenced for individual M100781. This individual has a biallelic expansion with the same 5' interruption composed of approximately 20 AAGGAG repeats (likely due to distant consanguinity). **B)** Schematic representation of nanopore reads sequenced for family E24-0752: the two affected family members have intermediate *FGF14* alleles with 203 and 207 repeats, respectively. Three of the offspring received an expanded allele that contracted (154, 164, and 164 repeats, respectively) during the paternal meiosis. Blue: AAG repeats; Yellow: GAG; Red: AGG; Green: ACG; Pink: AAC; Black: other.

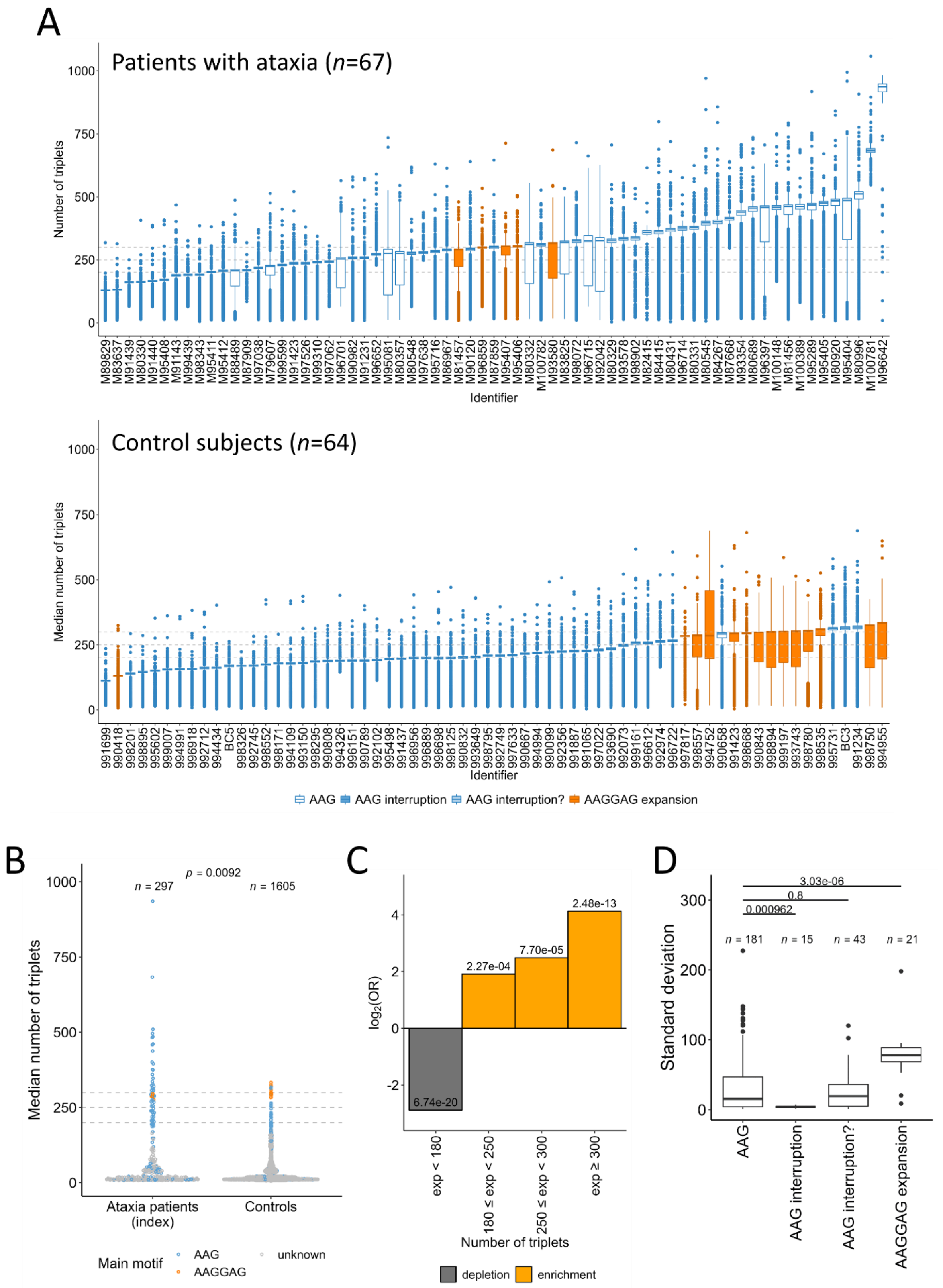

Supplementary Figure 5. **Distribution of *FGF14* alleles in patients with cerebellar ataxia and control subjects. A)** Box plots showing the distribution of the number of triplets on both alleles calculated from nanopore data for the 67 patients with ataxia and 64 control individuals sequenced by nanopore sequencing. Pure AAG alleles are depicted in blue with a white fill;

AAGGAG alleles in orange. Alleles with interruptions are depicted in blue with a dark blue fill. Alleles with interruptions limited to the 5’ or 3’ of the expansion are depicted in blue with a light blue fill. Box plot elements are defined as follows: center line: median; box limits: upper and lower quartiles; whiskers: 1.5× interquartile range; points: outliers. **B)** Comparison of the median sizes (all alleles; Mann-Whitney U test, two-sided) in patients with cerebellar ataxia and control subjects. Blue: AAG; orange: AAGGAG; gray: unknown main motif. **C)** Log odds ratio according to repeat numbers (ataxia patients versus controls) showing a significant enrichment of alleles > 180 repeats in patients with cerebellar ataxia (Fisher’s tests, two-sided, adjusted for multiple comparisons using Bonferroni correction; yellow: enrichment; gray: depletion). Each bar represents a single data point. **D)** Standard deviation as a measure of somatic variability for alleles with pure AAG repeats, alleles with interruptions limited to 3’ or 5’, true interruptions (disrupting repeats), and alleles with AAGGAG repeats. Box plot elements are defined as follows: center line: median; box limits: upper and lower quartiles; whiskers: 1.5× interquartile range; points: outliers. Comparisons were performed by applying Mann-Whitney U test, two-sided, followed by Holm correction for multiple testing.

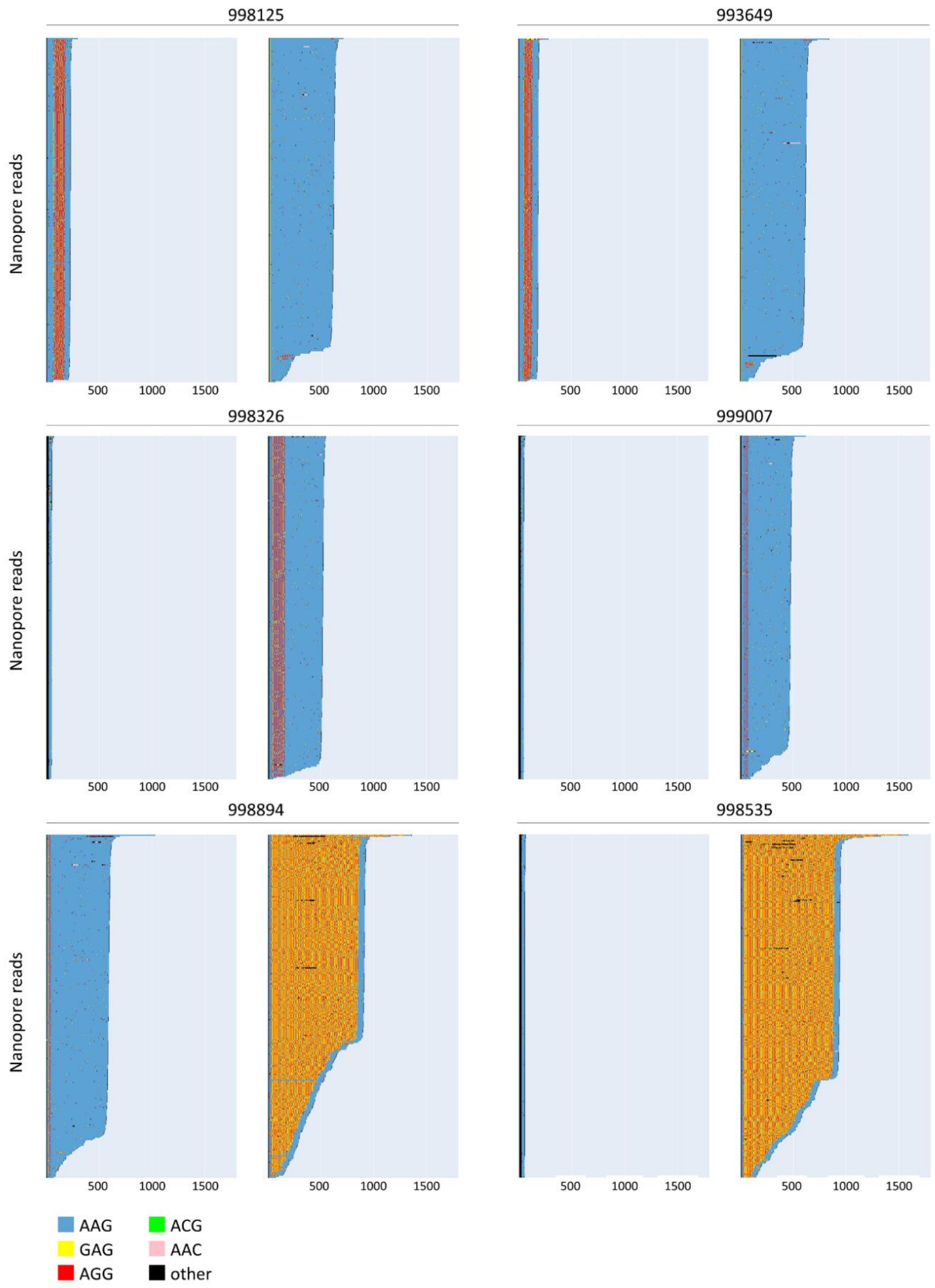

Supplementary Figure 6. Examples of nanopore read profiles detected in control subjects. This figure shows examples of true AAG interruptions in small alleles (2 panels above), interruptions limited to 3' or 5' sides of the repeats in large alleles (2 panels in the middle), and large alleles mainly composed of another hexameric (AAGGAG) motif (2 panels below). Blue: AAG repeats; Yellow: GAG; Red: AGG; Green: ACG; Pink: AAC; Black: other.

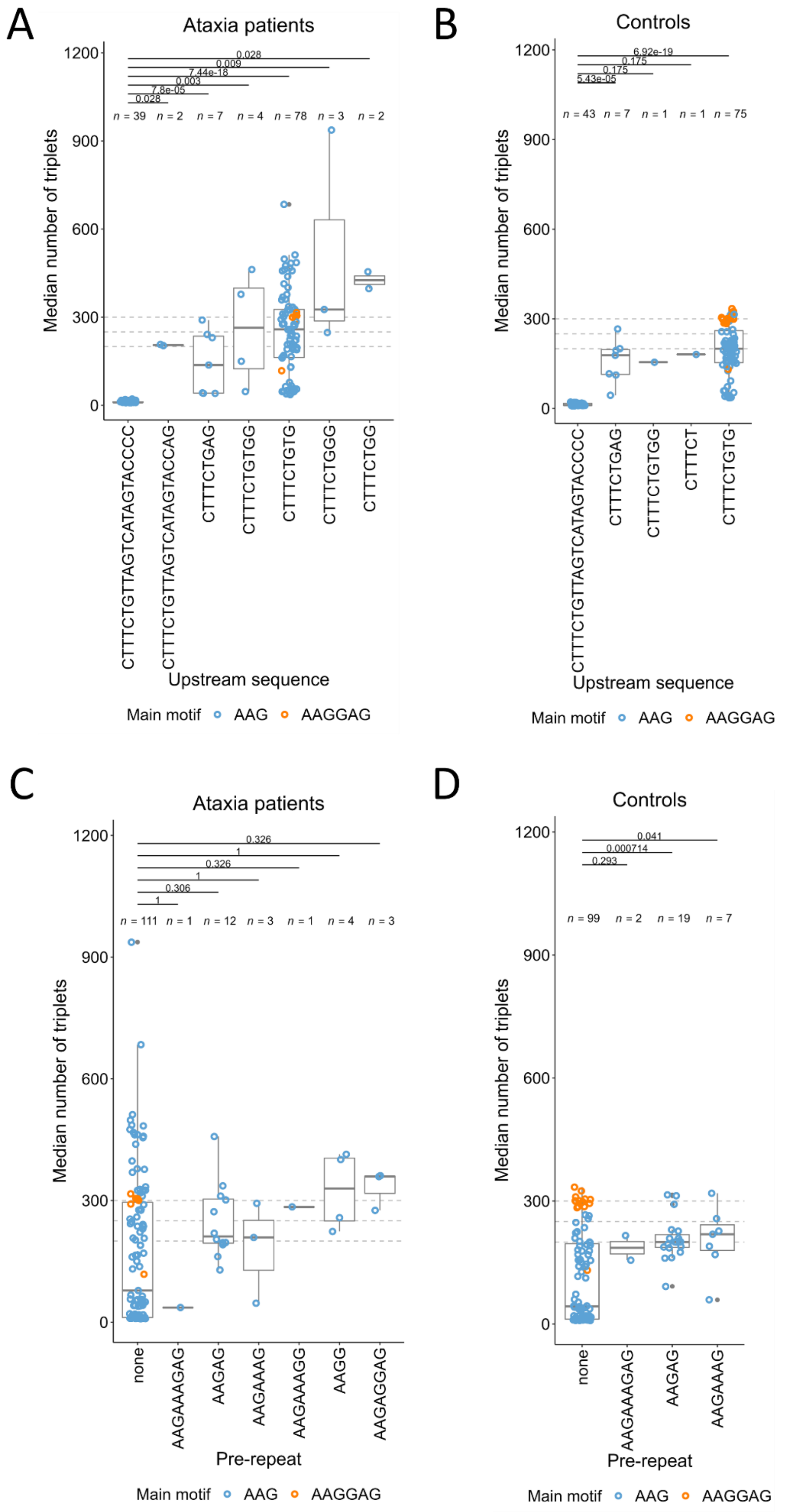

Supplementary Figure 7. Effect of 5' flanking regions on repeat instability. A) and **B)** Median number of triplets for each allele according to the flanking region sequence in patients

with ataxia (A) and in control subjects (B). **C)** and **D)** Median number of triplets for each allele according to the pre-repeat motif in patients with ataxia (C) and in control subjects (D). Box plot elements are defined as follows: center line: median; box limits: upper and lower quartiles; whiskers: 1.5× interquartile range; points: outliers. Comparisons were performed by applying Mann-Whitney U test, two-sided, followed by Holm correction for multiple testing. Blue: AAG; orange: AAGGAG main motif.

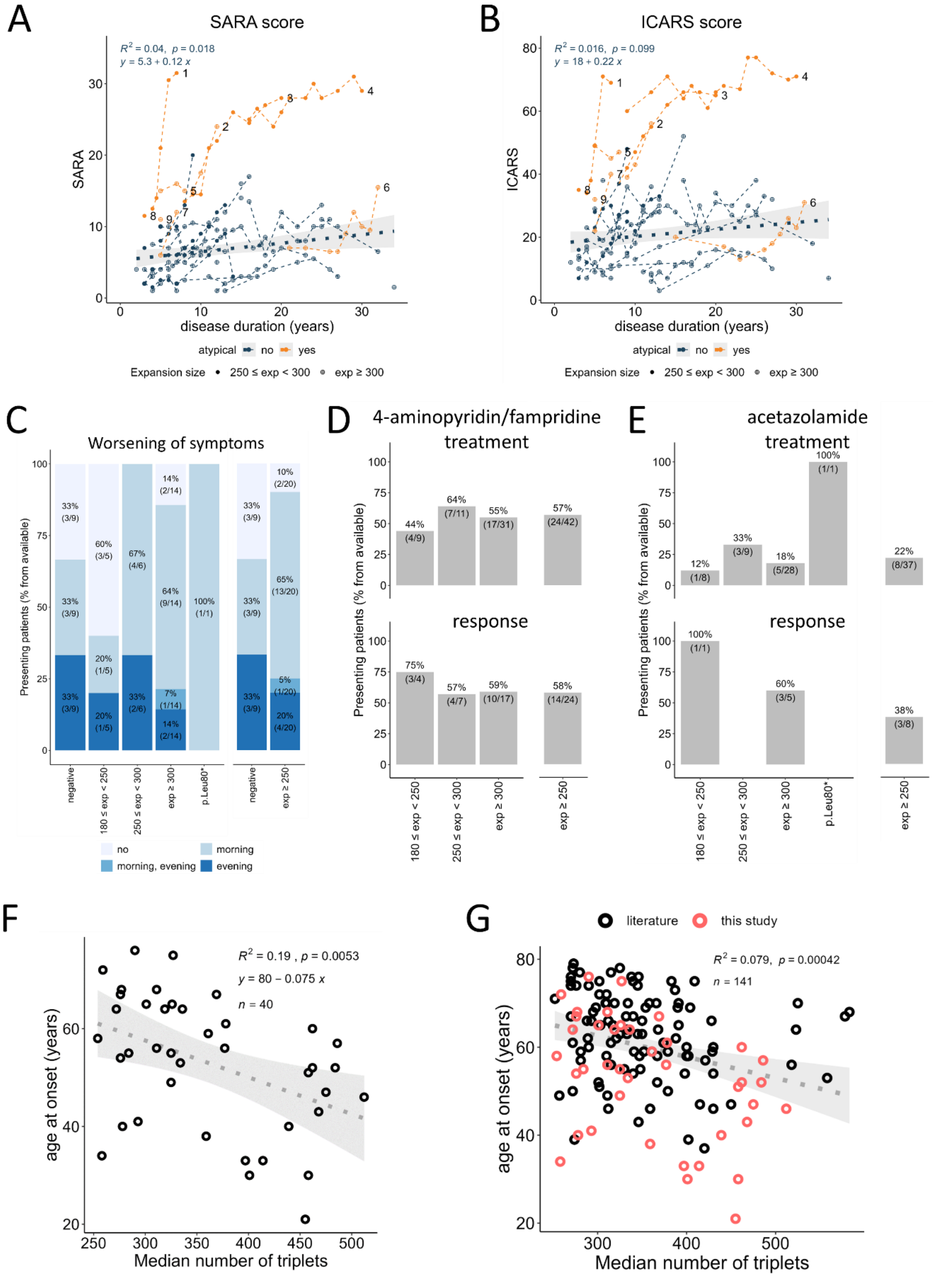

Supplementary Figure 8. **Additional clinical comparisons**. **A)** SARA scores of patients with *FGF14* repeat expansions. **B)** ICARS scores of patients with *FGF14* repeat expansions. In both graphs shown in panels E) and F), atypical patients appear in orange while the other appear in

blue. Scores from the same patients at different time points are connected with dashed lines. Numbered last data points mark lines corresponding to atypical patients (#1-6) or patients with biallelic expansions (#1-3 and #7-9). SARA and ICARS scores are clinical rating scales that are used for semi-quantitative assessment of cerebellar ataxia (see methods). **C)** Bar graphs showing the percentage of patients presenting a worsening of symptoms on morning (medium-light blue), evening (dark blue), morning and evening (medium-dark blue) or no worsening of symptoms depending on the day time (light blue). **D)** Bar graphs showing the response to 4-aminopyrimidine/fampridine treatment in patients with intermediate alleles, patients with 250 to 299 repeats and patients with ≥ 300 repeats (left). On the right, the graph shows the response for all patients ≥ 250 repeats. **E)** Bar graphs showing the response to acetazolamide treatment in patients with intermediate alleles, patients with 250 to 299 repeats, and patients with ≥ 300 repeats (left). On the right, the graph shows the response for all patients ≥ 250 repeats. **F)** Correlation between the age at onset and *FGF14* AAG repeat number including only patients from this study, excluding outliers. **G)** Correlation between the age at onset and *FGF14* AAG repeat number taking all patients from this study (red) and patients from previous studies (black) into account, excluding outliers. For graphs A), B), F), and G), R^2 is the square value of the Pearson correlation coefficient (two-sided) and 95% confidence intervals appear in light gray. For graphs C) to E), each bar represents a single aggregated data point (in percentage).

Supplementary case reports

Patients #1-6: clinical outliers ; Patients #1-3 have biallelic expansions
Patients #7-9: patients with biallelic expansions and typical disease course

M95716;#1 (E20-0778)

This patient was referred at the age of 59 years because of an unexplained cerebellar syndrome with a progressive gait disturbance persisting for approximately 3-4 years. Initial symptoms started at age 55, characterized by leg stiffness accompanied by pain and a propensity to fall. Subsequently, the patient developed diplopia and ascending numbness of the extremities. The initial neurological examination revealed a cerebellar syndrome with moderate dysarthria, cerebellar oculomotor disorder featuring downbeat nystagmus, and hypoesthesia of the lower legs and forearms. As part of an inpatient assessment, there was evidence of borderline pleocytosis (5 leucocytes/µl), type 2 oligoclonal bands in cerebrospinal fluid (CSF), elevated antinuclear antibody (ANA) titers (1:640), and the presence of anti-TPO and anti-Tg antibodies. Brain MRI indicated incipient vermal cerebellar atrophy, with no detectable inflammatory lesions in the central nervous system (CNS), including spinal MRI. Tumor screening including FDG-PET/CT, and screening for antineuronal/onconeural antibodies yielded normal results. Type 2 oligoclonal bands were considered unspecific. Electrophysiological examinations (nerve conduction studies, sensory evoked potentials) revealed no signs of polyneuropathy or dorsal column involvement. Throughout the disease course, cerebellar symptoms markedly intensified (19 points over 3 years in SARA score), leading to the patient's inability to stand or walk even with strong support. Additionally, there was a development of slightly increased tone in the legs and increase muscle reflexes, indicative of pyramidal tract involvement. Genome sequencing including identified a heterozygous splice variant in *ALDH18A1* (NM_002860.4:c.558+1G>A) and a heteroplasmic variant (m.14484T>C; p.(Met64Val) in the mitochondrial *MT-ND6* gene. Loss-of-function variants in *ALDH18A1* are typically recessive and no second variant was identified in the gene. Furthermore, proline, ornithine and citrulline levels, which are typically reduced in SPG9A/B, were normal. The p.(Met64Val), although classified as pathogenic in ClinVar, is associated with a specific haplogroup (haplogroup J) and occur in association with other variants in the context of Leber hereditary optic neuropathy (LHON). These two variants are unlikely to be responsible alone for the patient’s phenotype but could contribute to the atypical presentation. This study revealed a biallelic (196/284) AAG repeat expansion in *FGF14*.

M80332;#2 (E19-1058)

This patient was referred at the age of 65 years because of a progressive gait disorder. About 9 years prior to presentation, gait instability had been noticed for the first time, which was initially attributed to a concomitant chronic pain disorder and a phobic component with fear of recurrent falls. In 2014, the patient underwent surgery for spinal canal stenosis (LWK4/5) and a herniated disc (LWK5/S1), which did not lead to any improvement in her symptoms. Due to the anxiety disorder and a recurrent depressive disorder, she underwent repeated inpatient psychiatric treatments. Brain MRI scans showed mild vermal cerebellar atrophy. On initial examination, cerebellar ataxia with cerebellar oculomotor dysfunction, ataxia of stance and gait, and limb ataxia was found. Walking was only possible with substantial support for a few meters, and in daily life, the patient was already reliant on a wheelchair. There was a pronounced fear of falling. An older brother also suffered from stance and gait unsteadiness. No similar symptoms were reported for the parents, with the father having passed away at the age of 66 years. Subsequently, the younger sister also developed a cerebellar syndrome. Given

the family history, hereditary ataxia was suspected, leading to the initiation of molecular genetic testing, which excluded SCA1, 2, 3, 6, 8, 10, 11, 12, 17, 19, 23, 26, 34, 35, 48, DRPLA, Friedreich ataxia, Fragile X-Associated Tremor/Ataxia syndrome, and RFC1-CANVAS. An ataxia panel also yielded no abnormalities. The cerebellar syndrome progressed over three years (increasing by 9 points in the SARA Score). A mild left hemispheric ischemia (NIHSS 2 points) that had occurred in the meantime did not appear to have any influence on the cerebellar symptoms with a complete regression of symptoms. Within the scope of the EXPAND study, a biallelic expansion in the *FGF14* gene (311/204 repeats) was subsequently confirmed as the cause of the symptoms, with the anxious-depressive syndrome likely contributing.

M97638;#3 (E16-0360)

This patient was referred at the age of 48 years because of a slowly progressive cerebellar syndrome, characterized by instability of stand and gait, fine motor impairment, and dysarthria. The first symptoms occurred 8 years prior to presentation. Approximately 4 years after the onset of symptoms, a surgical decompression was performed at the level of C5/C6 due to a herniated disc with spinal canal stenosis, which had no effect on the gait disorder. Further external assessment of the symptoms revealed bihilary lymphadenopathy, granulomatous changes in a muscle biopsy and an elevated angiotensin-converting enzyme level, leading to the assumption of sarcoidosis with muscular involvement as the cause of the symptoms. A temporary corticosteroid therapy was initiated. With increasing cerebellar symptoms and a positive family history (similar symptoms in the father and paternal grandmother), autosomal-dominant hereditary ataxia was assumed. On initial examination, a cerebellar syndrome without extracerebellar involvement was found, in particular no evidence of muscular involvement. In the molecular genetic examinations, SCA1,2,3,6,7,8,11,12,13,14,15,17, 27A, episodic ataxia type 2, SPG7, and Friedreich's ataxia were excluded. In an ataxia panel with 133 ataxia-associated genes, two variants of unclear significance (VUS class 3) were found in the *NPHP1* (NM_001128178.3:c.232T>C) and *SPTBN2* gene (NM_006946.4:c.1456G>A), which were considered as unlikely to contribute to the phenotype. The patient developed moderate cerebellar atrophy over the course of the disease. The cerebellar symptoms markedly increased over the course of the disease (13.5 points over 12 years in the SARA score). This study showed a biallelic (278/222) AAG repeat expansion in *FGF14*.

M90120;#4 (E22-0036)

This patient was first seen at the age of 50 years because of cerebellar ataxia, which had been known for 9 years. The patient had been suffering from epilepsy since the age of 24 years, and had undergone several operations on an epidermoid tumor of the basal cisterns (at the age of 37, 39 and 44 years). For the treatment of her epilepsy, she received phenytoin until the age of 37 years (i.e. first surgery). She also suffered from migraine. The initial examination revealed marked cerebellar ataxia with cerebellar oculomotor dysfunction and severe ataxia of stance and gait. The patient was unable to stand without assistance. Brain MRI scans revealed significant cerebellar atrophy, along with residual epidermoid tumor, falx meningioma and a right temporolateral meningioma. As family history and previous inpatient investigations remained unremarkable, the ataxia was initially classified as sporadic adult-onset ataxia of unknown etiology (SAOA). In the molecular genetic tests, SCA1, 3, 6, and 7 were excluded, an exome-based ataxia panel showed negative results. Annual MRI examinations revealed a progressive meningioma of the posterior fossa and increasing leukoencephalopathy. The patient also developed dementia over time. The cerebellar symptoms progressed only slowly (albeit at a high level) in the following years (4 points over 14 years). *FGF14* analysis revealed a heterozygous expansion (293 repeats) in *FGF14*.

M81456;#5 (E20-0198)

This patient was referred at the age of 57 years because of a progressive cerebellar syndrome, which had been present for at least 5 years. Given the positive family history (including 4 out of 7 siblings, the maternal mother, and maternal grandmother with similar symptoms), there was suspicion of autosomal-dominant hereditary ataxia. The initial clinical neurological examination revealed a pronounced cerebellar syndrome with downbeat nystagmus. Unlike other affected family members, the patient also presented with choreiform hyperkinesia and increased muscle tone of the lower limbs. There were episodic exacerbations of both hyperkinesia and cerebellar symptoms. During a hospital stay for further phenotyping, no evidence of (additional) extracerebellar involvement was found. Molecular genetic investigations (conducted on the patient and her sister) yielded unremarkable results, including SCA1, 2, 3, 6, 7, 8, 10, 12, 14, 17, 37, *RFC1, HTT, ATN1*, and *C9orf72*. Brain MRI revealed vermian cerebellar atrophy. The patient reported improved gait instability with fampridine treatment. A probatory therapy with tiapride had a positive effect on the cerebellar oculomotor disorder (oscillopsia), but not on the hyperkinesia, and was stopped over time. Hyperkinesias improved with olanzapine administration. The clinical course has remained relatively stable over the three years since the initial presentation. This patient has a heterozygous expansion (462 repeats) in *FGF14*.

M80920;#6 (E20-0040)

This patient was referred at the age of 62 years because of a slowly progressive balance disorder that had been present for approximately 10 years. Initial examination revealed no abnormalities except for a downbeat nystagmus. As an incidental finding, Duane syndrome was present. Over time, the patient developed a mild pancerebellar syndrome that only mildly worsened over approximately 20 years. In addition, episodic worsening of balance was reported, lasting for one to two hours, and decreasing in severity over the years. Brain MRI scans showed a slowly progressive atrophy of the cerebellar vermis. Genetic analysis gave no indication of spinocerebellar ataxias type 1, 2, 3, 6, 7, 8, 10, 12, and 17, Friedreich's ataxia, *RFC1* (CANVAS), and Fragile X Tremor-Ataxia Syndrome. In the course of the disease, the patient also developed autonomic dysfunction (orthostatic hypotension, erectile dysfunction, urinary symptoms), and around the age of 80, and Parkinson's syndrome beginning with resting tremor in the left hand and with initial responsiveness to levodopa, and later in the course dementia. Except for the last SARA/ICARS score, there was no definite influence of the Parkinson's syndrome on the clinical ataxia scores. A benign form of multiple system atrophy (MSA-C) or the presence of two diseases (cerebellar disease and idiopathic Parkinson’s syndrome) were discussed as possible differential diagnoses. A heterozygous pathogenic expansion (484 repeats) was identified in *FGF14*.

M83825;#7 (E19-1058)

This patient (brother of case M80332;#4) was first seen at the age of 69 years because of a progressive gait disorder. About 5 years prior to presentation (after prostate surgery), gait instability with exacerbation after the intake of even small amounts of alcohol had been noticed for the first time. A brain MRI scan showed cerebellar atrophy, which was most pronounced in the vermis. The initial clinical neurological examination revealed a mild cerebellar syndrome with downbeat nystagmus and additional signs of polyneuropathy. Given the positive family history (two younger sisters with similar symptoms) hereditary ataxia was suspected. Due to the extensive genetic testing of his sister (see case report#4), no further genetic testing was carried out. The cerebellar syndrome progressed over two years (increasing by 6 points in the SARA Score). A bilateral total knee replacement (surgery in 2010 and 2018) did not appear to have any influence on the cerebellar symptoms. Within the scope of the EXPAND study, a

biallelic expansion (319/196 repeats) in *FGF14* was subsequently confirmed as the cause of the symptoms.

M96652;#8 (E23-0439)

This patient was referred at the age of 67 years because of a slowly progressive balance disorder that had been present for approximately 3 years. For six months, the symptoms only occurred episodically for a few minutes, but since then were persistent and particularly pronounced in the morning. Episodic worsening still occurred about three times a week, lasting about 15-20 minutes and could be triggered by even small amounts of alcohol or caffeine. Dysarthria and an urge incontinence were also reported. A brain MRI showed a mild cerebellar atrophy. Initial examination revealed a cerebellar syndrome with a severe cerebellar oculomotor disorder and subtle dysarthria. The patient reported improved gait instability and less severe episodes with fampridine treatment.Based on the positive family history (the mother developed similar symptoms around the age of 75 years) autosomal-dominant hereditary ataxia was assumed and genetic testing was recommended. A biallelic repeat expansion (272/165 repeats) in *FGF14* was discovered in the context of the EXPAND study.

M100781;#9 (E24-0221)

This was referred at the age of 37 years because of an unexplained cerebellar syndrome, which had been present for at least 5 years. Initially, symptoms only occurred episodically, lasting for several minutes up to two hours, but became persistent over time. Since the examination results were unremarkable between episodes and the molecular genetic testing for episodic ataxia (exome sequencing) remained inconclusive, a functional movement disorder was suspected, leading to recommendations for psychiatric or psychotherapeutic evaluation. The initial neurological examination in our outpatient clinic revealed a cerebellar syndrome with a cerebellar oculomotor disorder without downbeat-nystagmus, ataxia of stance and gait, and dysarthria. The family history remained unremarkable, although consanguinity between the parents, both from a small village in a Middle Eastern country, could not be definitively ruled out. Brain MRI showed cerebellar atrophy and attenuated parietal brain atrophy. The patient also reported a temporary improvement in symptoms during a previous treatment with acetazolamide. A very large biallelic repeat expansion with the same 5’interruptions in *FGF14* (684/498 repeats) was identified in the scope of the EXPAND study. This patient is the only affected of 9 children, suggesting a recessive inheritance of this expansion configuration. Unfortunately, other family relatives were not available for genetic testing at the time of this study.
