# Supplementary material for: Identification and characterisation of pathogenic and non-pathogenic FGF14 repeat expansions
Source: Nat Commun. 2024 Sep 3;15:7665. doi: 10.1038/s41467-024-52148-1 (PMC11372089; doi:10.1038/s41467-024-52148-1)
Supplement: Supplementary file 3 — Description of Additional Supplementary Files [file 41467_2024_52148_MOESM3_ESM.pdf]

Description of Additional Supplementary Files

File Name: Supplementary Data 1-8

Description: Excel file containing Supplementary Data 1-8 in individual sheets:

Supplementary Data 1. Comprehensive overview of cohort composition and genetic findings

Supplementary Data 2. Genetic data

Supplementary Data 3. qPCR experiments showing that individual M82415 has two copies of FGF14

Supplementary Data 4. Statistical test results

Supplementary Data 5. Detailed clinical data

Supplementary Data 6. Age at onset and FGF14 variant class information for the meta-analysis

Supplementary Data 7. Mean AAO stratififed according to expansion size (meta-analysis)

Supplementary Data 8. Number of repeats corresponding to the 85th, 90th, 95th and 99th quantiles detected by ExpansionHunter (with and without off-targets) and STRipy extended
