# Supplementary material for: Identification and characterisation of pathogenic and non-pathogenic FGF14 repeat expansions
Source: Nat Commun. 2024 Sep 3;15:7665. doi: 10.1038/s41467-024-52148-1 (PMC11372089; doi:10.1038/s41467-024-52148-1)
Supplement: Supplementary file 5 — Reporting Summary [file 41467_2024_52148_MOESM5_ESM.pdf]

nature portfolio

Corresponding author(s): Christel Depienne

Last updated by author(s): Jul 10, 2024

Reporting Summary

Nature Portfolio wishes to improve the reproducibility of the work that we publish. This form provides structure for consistency and transparency in reporting. For further information on Nature Portfolio policies, see our Editorial Policies and the Editorial Policy Checklist.

Statistics

For all statistical analyses, confirm that the following items are present in the figure legend, table legend, main text, or Methods section.

n/a	Confirmed	
☐	☒	The exact sample size (*n*) for each experimental group/condition, given as a discrete number and unit of measurement
☒	☐	A statement on whether measurements were taken from distinct samples or whether the same sample was measured repeatedly
☐	☒	The statistical test(s) used AND whether they are one- or two-sided<br>*Only common tests should be described solely by name; describe more complex techniques in the Methods section.*
☐	☒	A description of all covariates tested
☐	☒	A description of any assumptions or corrections, such as tests of normality and adjustment for multiple comparisons
☐	☒	A full description of the statistical parameters including central tendency (e.g. means) or other basic estimates (e.g. regression coefficient) AND variation (e.g. standard deviation) or associated estimates of uncertainty (e.g. confidence intervals)
☐	☒	For null hypothesis testing, the test statistic (e.g. *F*, *t*, *r*) with confidence intervals, effect sizes, degrees of freedom and *P* value noted<br>*Give P values as exact values whenever suitable.*
☒	☐	For Bayesian analysis, information on the choice of priors and Markov chain Monte Carlo settings
☒	☐	For hierarchical and complex designs, identification of the appropriate level for tests and full reporting of outcomes
☐	☒	Estimates of effect sizes (e.g. Cohen's *d*, Pearson's *r*), indicating how they were calculated

Our web collection on statistics for biologists contains articles on many of the points above.

Software and code

Policy information about availability of computer code

Data collection	For gene fragment analysis and nanopore data acquisition, we used the Genemarker (SoftGenetics) and the MinKNOW (Oxford Nanopore Technologies) softwares, respectively.
Data analysis	Custom scripts developed and used for nanopore data analysis in this study are available in a Github repository.

For manuscripts utilizing custom algorithms or software that are central to the research but not yet described in published literature, software must be made available to editors and reviewers. We strongly encourage code deposition in a community repository (e.g. GitHub). See the Nature Portfolio guidelines for submitting code & software for further information.

Data

Policy information about availability of data

All manuscripts must include a data availability statement. This statement should provide the following information, where applicable:

- Accession codes, unique identifiers, or web links for publicly available datasets
- A description of any restrictions on data availability
- For clinical datasets or third party data, please ensure that the statement adheres to our policy

All data and scripts included in this study, with the exception of genome data, are available from the Supplementary tables or in the Github / Zenodo repositories. The consent forms of the EXPAND study do not allow depositing genome data in a public repository.

Research involving human participants, their data, or biological material

Policy information about studies with human participants or human data. See also policy information about sex, gender (identity/presentation), and sexual orientation and race, ethnicity and racism.

Reporting on sex and gender	This study includes patients with neurological disorders and control subjects that were recruited independently of their sex (or gender). The sex of the patients appear in Supplementary Data and in pedigrees (as sex is known to influence the expansion or contraction of FGF14 alleles). Sex of control individuals is not indicated as this is a not a variable considered in the analysis.
Reporting on race, ethnicity, or other socially relevant groupings	This study includes patients with neurological disorders and control subjects that were recruited in Germany or Spain, independently of their physical characteristics, skin color, ethnicity or other socially relevant grouping.
Population characteristics	This study includes patients with neurological disorders remaining unsolved after panel, exome or genome analysis seen in outpatient clinics in Germany and Spain who agreed to participate to this study. The age at examination or age at onset was not part of the criteria used to include patients although most had an adult-onset.
Recruitment	Patients were recruited based on the presence of a neurological disorder that remained genetically undiagnosed at the time of the study and their willingness to participate to the study.
Ethics oversight	The EXPAND study has received the approval of the ethics committee of University Hospital Essen (21-10155-BO).

Note that full information on the approval of the study protocol must also be provided in the manuscript.

Field-specific reporting

Please select the one below that is the best fit for your research. If you are not sure, read the appropriate sections before making your selection.

☒ Life sciences ☐ Behavioural & social sciences ☐ Ecological, evolutionary & environmental sciences

For a reference copy of the document with all sections, see nature.com/documents/nr-reporting-summary-flat.pdf

Life sciences study design

All studies must disclose on these points even when the disclosure is negative.

Sample size	We included in the study all patients available in our institute who had genetic data available. We had received DNA of approx. 900 control individuals from the HNR study but only 802 could be effectively amplified and used for the study.
Data exclusions	Only DNA of control individuals or patients that could not be amplified due to low quality were excluded from the analysis.
Replication	Each nanopore run includes the same positive and negative samples and we ensured that the values obtained did not differ between runs.
Randomization	not applicable
Blinding	Analysis was performed with numerical identifiers. The analysis of data was made by a person not aware of the clinical characteristics of the subjects.

Reporting for specific materials, systems and methods

We require information from authors about some types of materials, experimental systems and methods used in many studies. Here, indicate whether each material, system or method listed is relevant to your study. If you are not sure if a list item applies to your research, read the appropriate section before selecting a response.

Materials & experimental systems

n/a	Involved in the study	
☒	☐	Antibodies
☒	☐	Eukaryotic cell lines
☒	☐	Palaeontology and archaeology
☒	☐	Animals and other organisms
☐	☒	Clinical data
☒	☐	Dual use research of concern
☒	☐	Plants

Methods

n/a	Involved in the study	
☒	☐	ChIP-seq
☒	☐	Flow cytometry
☒	☐	MRI-based neuroimaging

Clinical data

Policy information about clinical studies

All manuscripts should comply with the ICMJE guidelines for publication of clinical research and a completed CONSORT checklist must be included with all submissions.

Clinical trial registration	not applicable
Study protocol	not applicable
Data collection	Clinical data were collected from the medical records of the patient retrospectively by two of the coauthors.
Outcomes	We used SARA and ICARS scores as a measure of clinical severity. Other clinical features were systematically retrieved from medical records.

Plants

Seed stocks	*Report on the source of all seed stocks or other plant material used. If applicable, state the seed stock centre and catalogue number. If plant specimens were collected from the field, describe the collection location, date and sampling procedures.*
Novel plant genotypes	*Describe the methods by which all novel plant genotypes were produced. This includes those generated by transgenic approaches, gene editing, chemical/radiation-based mutagenesis and hybridization. For transgenic lines, describe the transformation method, the number of independent lines analyzed and the generation upon which experiments were performed. For gene-edited lines, describe the editor used, the endogenous sequence targeted for editing, the targeting guide RNA sequence (if applicable) and how the editor was applied.*
Authentication	*Describe any authentication procedures for each seed stock used or novel genotype generated. Describe any experiments used to assess the effect of a mutation and, where applicable, how potential secondary effects (e.g. second site T-DNA insertions, mosiacism, off-target gene editing) were examined.*
